# Prevention report of Germany’s National Prevention Conference – Goals and possibilities of the initial 2019 report

**DOI:** 10.17886/RKI-GBE-2017-088

**Published:** 2017-08-30

**Authors:** Stefanie Liedtke, Stefan Gravemeyer, Guy Oscar Kamga Wambo

**Affiliations:** 1 National Association of Statutory Health Insurance Funds (GKV-Spitzenverband), Department Prevention; 2 German Social Accident Insurance(DGUV), Department Safety and Health; 3 German Statutory Pension Insurance, Department Social Medicine and Rehabilitation

## Abstract

The Preventive Health Care Act Germany adopted in July 2015 defines that the institutions involved in the National Prevention Conference have to publish a prevention report on a four year basis. In the report they have to detail their efforts towards settings-based primary prevention and health promotion. This article outlines the legal requirements for the prevention report and the status of the concept for the first report in 2019.

## Legal background

On 19 February 2016, Germany’s National Prevention Conference (NPK), which was established in autumn 2015, adopted its initial federal framework recommendations on settings-based primary prevention and health promotion [1]. These recommendations refer to three life course goals which the institutions involved in the NPK (GKV-Spitzenverband, DGUV, Social Insurance for Agriculture, Forestry and Horticulture, German Statutory Pension Insurance) share: Grow up healthy, Living and working healthy and Healthy ageing. For all three of these goals the recommendations describe the priority fields of action and target groups. Moreover, the services that statutory health and accident insurers, pension funds as well as long-term care insurance funds contribute are outlined. Possibilities and requirements for cooperation are addressed as well.

The Preventive Health Care Act [2] determines that, every four years, the institutions involved in the National Prevention Conference are to document and evaluate their activities regarding the implementation of the federal framework recommendations in a prevention report.

The report has to be submitted to Germany’s Federal Ministry of Health which will attach a German government statement and then forward the report to the federal legislative bodies. The first report is due on 1 July 2019.

In accordance with Book V of the German Social Code (SGB, § 20d section 4), the report has to provide information focusing on the following questions:

► Experiences concerning the application of key sections of the Preventive Health Care Act (SGB V, §§ 20–20g)
► Expenditure of the various branches of social security represented in the NPK and of private health care insurance funds as far as they hold voting rights in the NPK (as intended from February 2017 onwards)
► Forms of service delivery
► Individuals reached
► Achievement of joint goals and target groups
► Experiences with quality assurance
► Experiences with collaboration in the implementation of services

Moreover, the report has to provide conclusions and recommendations to refine the spending guidelines in primary prevention and health promotion for the services of health insurance funds effective since 2016 (seven euro per year per insured person, including at least two euro each for services in everyday settings and workplaces (SGB V, §20 section 6).

Individual member organisations of the institutions involved in the NPK will provide the information required for the report. The Robert Koch Institute delivers relevant information from its health monitoring; federal states can provide input from their regional health reporting (SGB V, § 20d section 4).

## Concept development

Based on the statutory provisions, the National Prevention Conference is currently developing a concept for the goals, methodology and content of the initial 2019 prevention report. The NPK meeting in February 2017 will decide on a draft concept, which will be developed in greater detail by the end of 2017. Beside the institutions involved in the NPK, NPK members with advisory status (the state, federal states, municipal central organisations, social partners, Federal Employment Agency, patient advocacy organisations, and the Federal Association for Prevention and Health Promotion (BVPG) as the representative of the prevention forum in accordance with SGB V (§ 20e paragraph 2)), as well as the Association of Private Health Care Insurers, the Robert Koch Institute and a scientific board support the development process. The more detailed concept and its methodological implementation, as well as writing the final report will require additional external support.

Settings-based primary prevention and health promotion require coordinated action by numerous stakeholders on federal, federal state and municipal levels. In the prevention report, the NPK institutions therefore intend to detail not only their own corresponding efforts for the envisaged reporting period of 2017 but also the efforts by all other NPK stakeholders. In addition, they aim to include information on the usefulness and effectivity of settings-based primary prevention and health promotion. Health monitoring information delivered by the Robert Koch Institute will provide data on the health situation in Germany and on prevention needs and potentials. Specific federal state and municipal level aspects will also be taken into account.

## Conclusion

By publishing the National Prevention Conference’s 2019 prevention report, comprehensive information on settings-based prevention and health promotion in Germany will be available for the first time. The second report in 2023 can illustrate developments. NPK institutions and their member organisations will use the gained information to refine their activities in the fields of prevention and health promotion. Furthermore, the report is aimed to indicate how all other NPK stakeholders can refine their activities in terms of action in society as a whole.
